# Artemisiae Annuae Herba: from anti-malarial legacy to emerging anti-cancer potential

**DOI:** 10.7150/thno.115414

**Published:** 2025-06-20

**Authors:** Jing Sun, Junzi Xufeng, Zejing Qiu, Quan Gao, Zhiyu Zhu, Jun He, Shegan Gao, Xinbing Sui

**Affiliations:** 1 Department of General Surgery, the Zhejiang Provincial People's Hospital, Hangzhou, Zhejiang, China.; 2 Department of Medical Oncology, the Affiliated Hospital of Hangzhou Normal University, Hangzhou Normal University, Hangzhou, Zhejiang 310015, China.; 3 School of Pharmacy, Hangzhou Normal University, Hangzhou, Zhejiang 311121, China.; 4 Henan Key Laboratory of Microbiome and Esophageal Cancer Prevention and Treatment, Henan Key Laboratory of Cancer Epigenetics, The First Affiliated Hospital, College of Clinical Medicine, Henan University of Science and Technology, Luoyang, Henan, China.

**Keywords:** Artemisiae Annuae Herba, traditional Chinese medicine, malaria, cancer, derivatives

## Abstract

Modern medical approaches to cancer treatment face significant obstacles, including limited therapeutic options, narrow drug applicability, and rapid development of drug resistance. Consequently, re-evaluating traditional medicinal plants and natural compounds has emerged as a promising strategy to address this public health issue, particularly amid challenges in developing novel pharmaceuticals. Artemisiae Annuae Herba, a versatile natural drug renowned for its established efficacy against malaria and for other diverse pharmacological activities, is gaining recognition for its anti-cancer potential due to the unique structures and biological effects of its constituents. This review comprehensively outlines the major components of Artemisiae Annuae Herba and their reported anti-cancer activities, beginning with an examination of the molecular structures of the foundational components and an exploration of derivatives of these compounds. Furthermore, through an analysis of observed pharmacological effects, we systematically elucidate the multifaceted influence of Artemisiae Annuae Herba on cancerous tissues, including cell cycle arrest, apoptosis induction, non-apoptotic cell death induction, angiogenesis inhibition, tumor microenvironment remodeling, and immune modulation. Finally, we discuss the feasibility of Artemisiae Annuae Herba in cancer therapy as well as the challenges and unresolved issues that require further investigation. We also consider ways that new drug formulations and routes of administration might overcome these translational hurdles. By synthesizing existing research on applications of Artemisiae Annuae Herba to cancer therapy, this review underscores potentially innovative clinical approaches, ultimately paving the way for the discovery of effective anti-cancer drugs with far-reaching benefits.

## Introduction

The biological complexity of tumors has presented significant challenges to the treatment of cancer. In addition, increasing levels of drug resistance have made some existing cancer therapies inadequate. Despite the development and clinical application of novel drugs, many patients still experience disease progression and unfavorable outcomes despite exhausting all relevant anti-cancer medications and participating in cutting-edge clinical trials. The processes of investigating the mechanisms of tumorigenesis and developing novel drugs require highly innovative thinking and are often hindered by the element of chance. Moreover, the protracted drug development timeline, from initial research to clinical application, stands in stark contrast to the urgent demand for effective cancer therapies. Consequently, re-evaluating and extending the anti-cancer potential of existing drugs, particularly those derived from traditional herbal medicines, has gained considerable scientific and medical significance. This approach offers a potentially more rapid and accessible avenue for addressing the pressing need for improved cancer treatments.

The integration of Traditional Chinese Medicine (TCM), including TCM-related natural products, with Western medicine 1,2, has recently become an important aspect of comprehensive cancer treatments 3-5. Modern medical science researchers have noted that the anti-cancer effects of natural TCM products involve a complex array of mechanisms 6. Previously, the absence of advanced micro-analysis techniques hindered the ability of researchers to analyze underlying mechanisms and effects. However, advances in technical methods have now enabled molecular isolation, purification, and structural analyses to uncover novel insights 7-9. Given the existence of drugs or herbs with recognized anti-cancer properties, a comprehensive investigation is essential and promises to yield novel opportunities for cancer therapy.

Artemisiae Annuae Herba, a TCM herb derived from the plant *Artemisia annua L.* has a rich history 10 and has been demonstrated to hold a multifaceted therapeutic potential 11,12. It is effective against multiple conditions, such as malaria and rheumatism, and it exhibits emerging promise in cancer therapy 13. However, current research has predominantly focused on artemisinin and its derivatives 14-16, a component within the herb that gained attention for its anti-malarial properties, while neglecting the broader pharmacological actions of Artemisiae Annuae Herba's formulations and decoctions. It is worth noting that many researchers have recognized the potential of the herb's other constituents in cancer research and are actively investigating their effects. Despite challenges like purification complexity and interactions among the compounds, elucidating the mechanisms that underlie the anti-cancer activities of Artemisiae Annuae Herba and its components is a research avenue that promises to advance cancer treatment.

This review offers an in-depth analysis of Artemisiae Annuae Herba and its components or derivatives in cancer therapy, detailing their mechanisms, clinical applications, and usage considerations. In this review, we also identify critical research gaps and priorities to advance this field.

## The Basics of Artemisiae Annuae Herba

### Renaissance of ancient medicine: Artemisiae Annuae Herba

**Figure 1:**
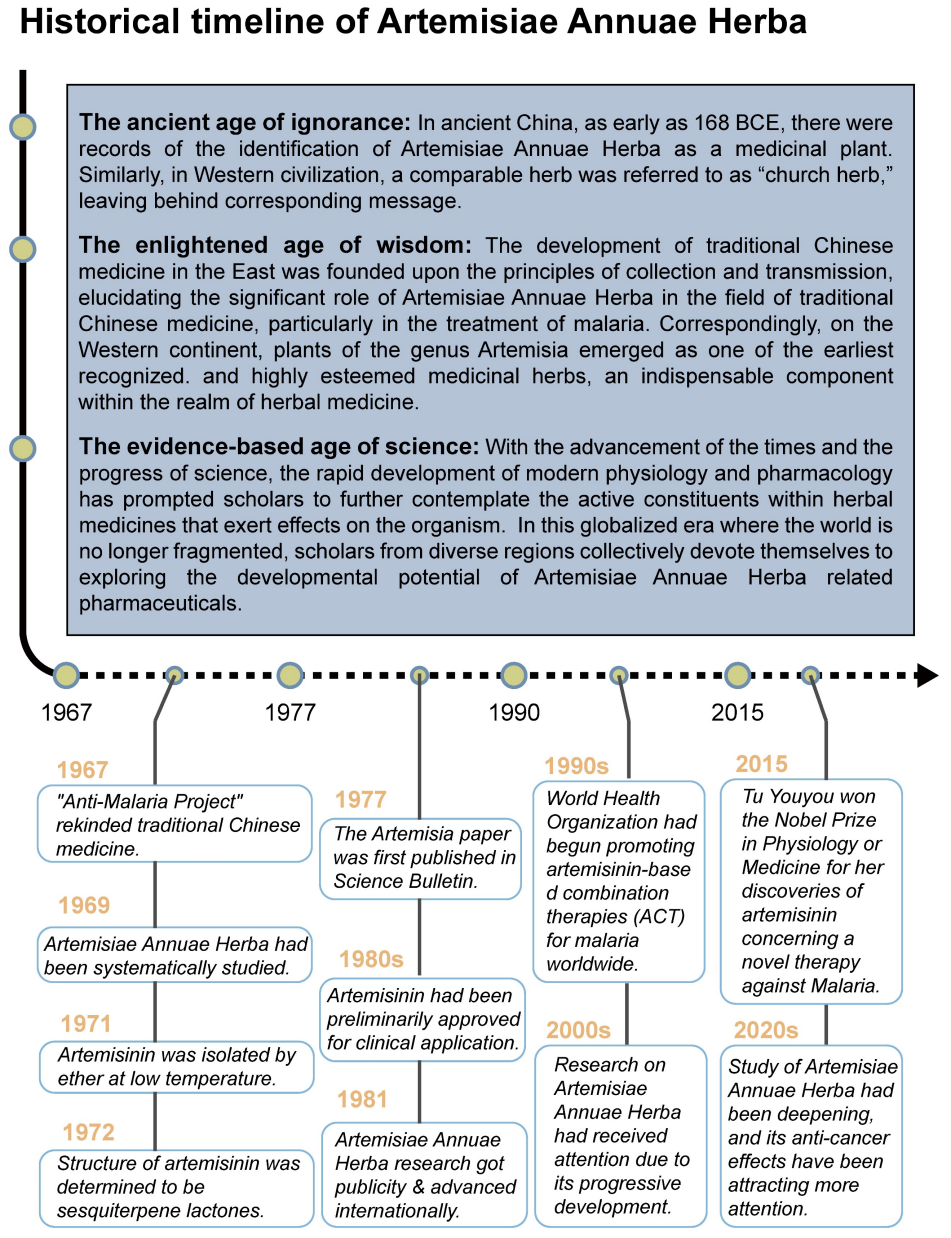
** Historical Timeline of Artemisiae Annuae Herba:** Development of Artemisiae Annuae Herba as medicine can be summarized into three periods: ancient age of ignorance, enlightened age of wisdom, and evidence-based age of science. Current research on Artemisiae Annuae Herba is rooted in the enlightenment era of scientific exploration and is moving forward steadily. In the 1960s, the drug was investigated intensely due to the need for anti-malaria treatments, and results were achieved in the 1970s and early 1980s. Other effects of the drug were considered after further promotion of it in the 1990s. Research of Artemisiae Annuae Herba entered the 21st century, with the Nobel Prize serving as a sign of progress.

The medicinal application of Artemisiae Annuae Herba has a long and rich history *(Figure 1)*, originating in ancient China. The Silk manuscript *Prescription for Fifty-two Diseases*, unearthed in the Mawangdui tomb (Changsha, Hunan Province, 168 BCE), documents one of its earliest medicinal uses, as part of a hemorrhoid treatment. Several other classical Chinese herbal medicine texts also provided detailed descriptions of the plant's therapeutic properties. For instance, *Shennong's Herbal Classic* (ca. 100 CE) characterizes Artemisiae Annuae Herba as “bitter and cold in taste,” with applications for treating scabies, itching, sores, lice infestations, and heat retention in the bones and for vision improvement. Similarly, *New Repair of Materia Medica* (ca. 659 CE) emphasizes its external use for “applying to sores, promoting hemostasis, regenerating tissue, and relieving pain.” These early records indicate that Artemisiae Annuae Herba was primarily employed for both internal and external treatments of skin infections, such as pruritus and scabies, with a secondary mention of its potential to kill lice and prevent insect-borne diseases.

In Western civilizations, a thorough definition of Artemisiae Annuae Herba emerged later, with the plant's original scientific name, *Artemisia annua L.*, being established in 1753 by Carl Linnaeus. The name *Artemisia* refers to the genus within the Asteraceae (chrysanthemum) family, and *annua* denotes its annual growth cycle. Despite the relatively recent adoption of its scientific nomenclature, the plant's reputation had already spread widely across various cultures. In the Arab world, *Artemisia annua L.* was valued for its ability to treat ulcers, skin ailments, and hair loss, and it even served as a talisman against evil. Among Slavic communities, it was termed the “church herb,” while in Poland, it was affectionately referred to as “God's little tree.” These diverse cultural associations underscore the plant's longstanding significance in traditional medicine and its symbolic importance globally 17.

As scholars' knowledge has progressed and technology has advanced, our understanding of Artemisiae Annuae Herba has deepened significantly. In Asia, particularly within the context of TCM, the efficacy of this herb has been continuously verified and extended. Ge Hong (283-343 CE), a philosopher and pharmacist of China's Eastern Jin Dynasty, played a crucial role in identifying and validating the use of Artemisiae Annuae Herba for malaria treatment. In *Handbook of Prescriptions for Emergency Treatment* (ca. 326-341 CE), he detailed its anti-malarial applications, with instructions to extract its juice by soaking in water. This foundational understanding was further developed by subsequent TCM practitioners. Song Dynasty scholar Zhou Qufei (1134-1189 CE), documented the use of Artemisiae Annuae Herba as a key treatment for malaria and other ailments in his book *Questions and Answers from Beyond Mountain* (ca. 1178 CE); the use of Artemisiae Annuae Herba was often combined with acupuncture and bloodletting to improve effectiveness. Meanwhile, in Western Europe, research into herbal medicine also explored the potential of other *Artemisia* species. The herb *A. abrotanum* (southernwood), a congener species, held a significant place in European traditional medicine 18. It was predominantly recommended for liver and biliary diseases and increasingly utilized as an efficient deworming and antipyretic agent for both adults and children. These parallel traditions in different regions underscore the global historical appreciation of *Artemisia's* therapeutic value.

The resurgence of interest in Artemisiae Annuae Herba in modern China aligns with the revitalization of TCM practices 19. Since 1967, many researchers in China have systematically investigated TCM through modern scientific methodologies. Among these pioneers 20-23, Tu Youyou stands out as a particularly notable figure. She achieved a significant breakthrough by isolating and extracting sesquiterpene lactones artemisinin from Artemisiae Annuae Herba. Her innovative approach involved using cold brewing and ingredient analysis 24,25, rather than the traditional hot decoction methods described in ancient texts. This advancement marked a pivotal moment in the treatment of malaria 26,27 and redefined the therapeutic use of Artemisiae Annuae Herba 28,29. Tu was awarded the 2015 Nobel Prize in Physiology or Medicine for this groundbreaking discovery 30.

Beyond its established anti-malarial properties, Artemisiae Annuae Herba has garnered considerable scientific interest for its varied therapeutic effects 31 and biochemical properties 32,33, especially concerning artemisinin 34-37. The anti-cancer potential of various components of Artemisiae Annuae Herba or their derivatives 38 has become a significant research focus. Since the 1990s, both Eastern and Western scientific communities have investigated the cytotoxicity of artemisinin and its derivatives 39,40, such as dihydroartemisinin (DHA) 41-43, artesunate (ART) 44-46, artemether (ARM) 47-50, and arteether (ARE) 51,52, across diverse cancer cell lines 53. The early studies initially confirmed the anti-cancer potential of Artemisiae Annuae Herba components *in-vitro* 54-56. In addition, an increasing number of *in-vivo* studies using mouse models of human xenograft tumors 57-59, along with clinical trials combining chemotherapy with Artemisiae Annuae Herba-derived drugs 60, are further substantiating the tumor-inhibitory effects of these compounds 61,62.

### Accurate identification of Artemisiae Annuae Herba

A retrospective of the historical research on Artemisiae Annuae Herba reveals a diversity of appellations and naming conventions 63. The plant from which authentic Artemisiae Annuae Herba is derived, most commonly known as *A. annua L.*, is primarily identified by the morphologies of its rhizomes and leaves. The cylindrical rhizomes, branching in the upper section, exhibit yellowish green to brownish-yellow surfaces with longitudinal ridges. They are slightly hard, easily fractured, and possess a pith in the center of the fracture surface. The dark green to brownish-green leaves are fragile and often curled. Intact leaves display a tripinnately dissected morphology, with rectangular to oblong segments and lobules covered in short hairs. The herb possesses a distinct, aromatic odor and a slightly bitter taste.

Despite these distinctive features, other plants' products have been mistakenly identified or referred to as Artemisiae Annuae Herba, leading to some confusion. By synthesizing information from both ancient texts and modern scientific literature, it becomes evident that the medicinal herb commonly referred to as *Qinghao* in traditional and contemporary contexts is indeed Artemisiae Annuae Herba. Specifically, this formulation refers to the dried above-ground parts of *A. annua L.* 64,65, rather than other related species within the Asteraceae family, such as *A. flavescens*. The clarification is essential for the precise identification and applications of Artemisiae Annuae Herba in medicinal and scientific research.

Beyond identifying *A. annua L.* as the definitive source of Artemisiae Annuae Herba, its transformation from a simple plant to a medicinal herb warrants attention. Unlike other herbs requiring complex processing, traditional Artemisiae Annuae Herba preparation involved air-drying *A. annua L.* after removing aged stems in autumn. Modern methods, however, utilize continuous solvent extraction and product recovery. This purification reduces interactions between the various components, and it also allows for in-depth exploration of single compounds, enabling researchers to thoroughly analyze pharmacological effects and applications, offering unparalleled advantages. Decades of comprehensive chemical and biological analyses have isolated various bioactive compounds from Artemisiae Annuae Herba 66-68, including sesquiterpenoids 69,70, flavonoids 70,71, coumarins 72, steroids 24, phenolics 73, purines 74, and lipids 75. To date, over 600 components have been characterized, with novel compounds still continuing to be discovered 69,76. Based on the chemical structures of these components, their active structures, mechanisms of action, and anti-cancer potential deserve further analysis and investigation.

### Biological characteristics of Artemisiae Annuae Herba

Artemisinin is the most famous of the many bioactive components in Artemisiae Annuae Herba. Extensive research on artemisinin continues to uncover novel applications and research directions. In addition, the growing diversity of research approaches has led to the production and application of a broad range of derivatives 77. Derivative compounds such as artesunate, artemether, DHA, and others have already become first-line treatments for malaria 78-81. The versatility of artemisinin derivatives in the treatment of cancer, viral infection, immunity, and parasitic infections, as compared to the parent component, underscores the importance of further research on Artemisiae Annuae Herba.

**Figure 2:**
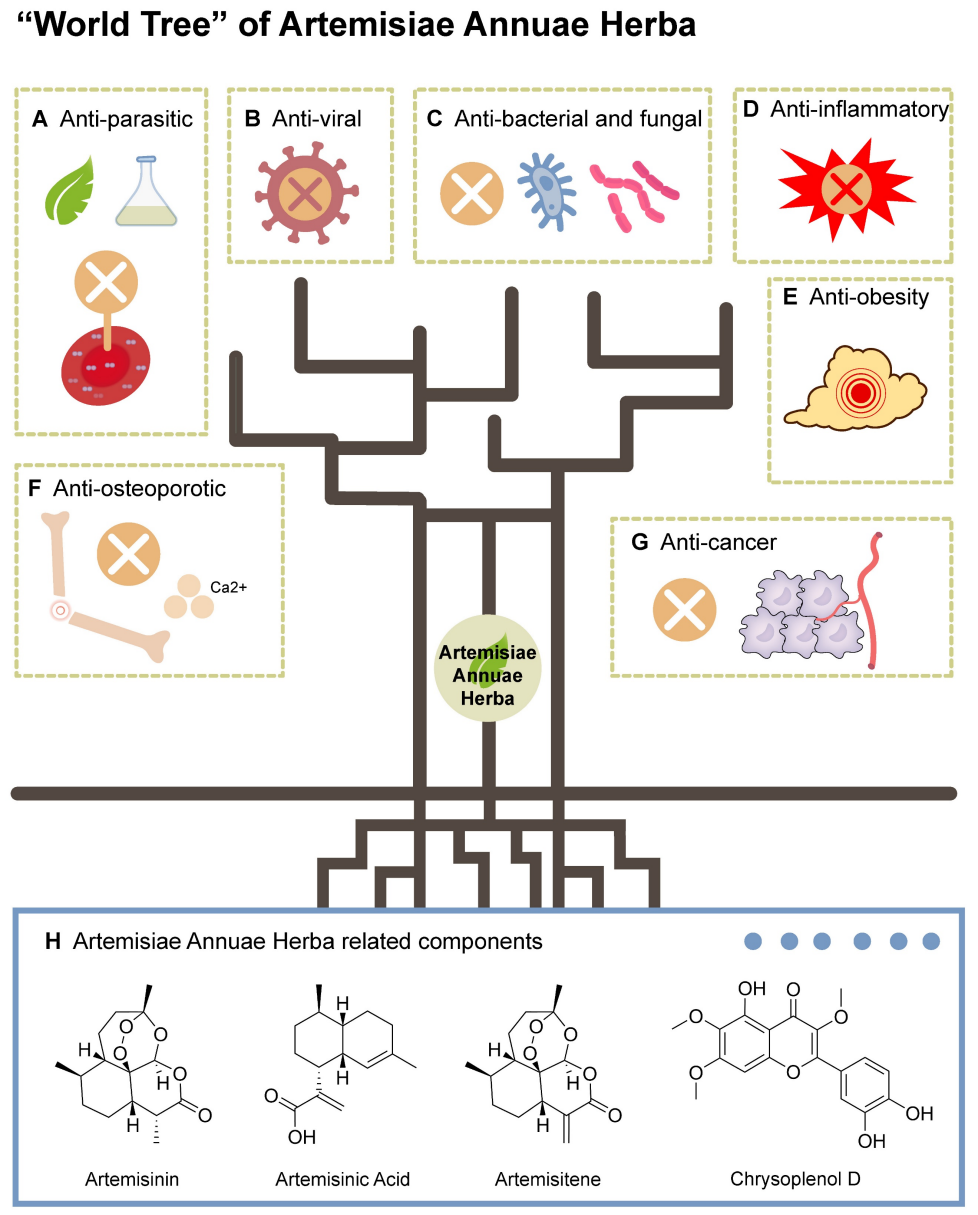
** “World Tree” of Artemisiae Annuae Herba:** Artemisiae Annuae Herba has a variety of components as its “roots,” and numerous biological activities and medicinal effects have been developed as its “branches and fruits.” Among the extracts of Artemisiae Annuae Herba, the main compounds are sesquiterpenoids, including artemisinin, artemisinic acid, and artemisitene, along with flavonoids, coumarins, steroids, phenolics, purines, and lipids. It has been developed for **A)** anti-parasitic, **B)** anti-viral, **C)** anti-bacterial and fungal, **D)** anti-inflammatory, **E)** anti-obesity, **F)** anti-osteoporotic, and **G)** anti-cancer applications.

Advancing research on Artemisiae Annuae Herba depends on the comprehensive elucidation of its biochemical effects as a pharmaceutical agent *(Figure 2)*. These effects can be categorized into several key activities: anti-parasitic, anti-viral, anti-bacterial and -fungal, anti-inflammatory, anti-obesity, anti-osteoporotic, and anti-cancer. The diverse therapeutic potential, especially the anti-cancer capability 13 of Artemisiae Annuae Herba has garnered increasing attention in the scientific community.

**Anti-parasitic:** Artemisiae Annuae Herba has been widely available and recommended for malaria prevention and treatment 82. Artemisinin, in particular, is most renowned for its anti-parasitic properties 83-85, particularly its efficacy against the causative agents of malaria, parasites of the genus *Plasmodium* 86-88. Malaria remains a leading cause of morbidity and mortality 89 in numerous countries 90,91, with infections varying from asymptomatic or mild to severe and potentially fatal. The World Health Organization has recommended Artemisinin-based Combination Therapies (ACTs) as the first-line treatment of uncomplicated *Plasmodium falciparum* malaria 92. Apart from malaria, artemisinin derivatives have also been shown to be effective against a number of other parasites, including *Toxoplasma gondii* 85, *Leishmania* 93,94, *Acanthamoeba* 84,95, and *Schistosoma* 96. Research has demonstrated the potent anti-parasitic effects of Artemisiae Annuae Herba components and derivatives, underscoring their therapeutic potential for various parasitic infections.

**Anti-viral:** Recent decades have brough substantial anti-viral research on artemisinin and derivatives, rather than the entirety of Artemisiae Annuae Herba. These compounds have exhibited efficacy against multiple viruses, including human herpesvirus 6, herpes simplex viruses 1 and 2, hepatitis B virus, and bovine viral diarrhea virus 10,97. Less anti-viral research has explored the activities of other Artemisiae Annuae Herba components, such as artemisinic acid, scopoletin, and arteannuin B, but as the understanding of Artemisiae Annuae Herba has deepened, and new challenging viruses like coronaviruses have arisen, other extracts from Artemisiae Annuae Herba have been found to demonstrate notable virucidal and anti-viral properties. Baggieri *et al.* 98 demonstrated that natural components extracted from Artemisiae Annuae Herba interact with 3-chymotrypsin-like protease and the spike protein from SARS-CoV-2. These natural components exert anti-SARS-CoV-2 activity by disrupting viral pathways during insertion and replication 99,100. The multiple anti-viral applications of Artemisiae Annuae Herba suggest that this herb is an important part of the clinical fight against viral infections.

**Anti-bacterial and fungal:** Recent studies have increasingly concentrated on the anti-bacterial and anti-fungal properties 101,102 of Artemisiae Annuae Herba, particularly its essential oils 103. These oils have demonstrated activities against diverse bacterial species 104-106, including both Gram-positive and Gram-negative bacteria, as well as fungi 101,107. The anti-microbial properties of Artemisiae Annuae Herba essential oils vary based on their geographical origin 102,108,109; variations in the chemical composition of oils between studies may account for the observed discrepancies. The essential oils of Artemisiae Annuae Herba exhibit significant variability in chemical composition, with camphor, artemisia ketone, and 1,8-cineole being the main anti-fungal and anti-bacterial agents 105. Elevated levels of these terpenoids correlate with enhanced anti-microbial activity 106,107. Overall, Artemisiae Annuae Herba holds promising potential as a source of novel anti-microbial agents. Systematic studies are required to comprehensively characterize the anti-microbial properties of Artemisiae Annuae Herba and to evaluate its advantages and limitations.

**Anti-inflammatory:** As the core, most intensely studied component, Artemisinin has been widely applied in various inflammatory disease contexts 110, such as autoimmune diseases, allergic inflammation, and sepsis. The anti-inflammatory effects of Artemisiae Annuae Herba have been attributed primarily to inhibition of MAPK and PI3K/AKT signaling pathways, activation of NF-κB, and modulation of the expression of the toll-like receptors TLR4 and TLR9 106. Beyond artemisinin, several other components of Artemisiae Annuae Herba have also exhibited anti-inflammatory properties 112,113, underscoring the significant therapeutic potential of Artemisiae Annuae Herba component-based interventions in inflammation management.

**Anti-obesity:** It is not appropriate to discuss the relationship between fat metabolism and components derived from Artemisiae Annuae Herba without reference to artemisinic acid, which has been shown to have highly anti-adipogenic activity *in-vitro* 114. Artemisinic acid has been shown to hinder adipogenic differentiation in adipose-derived mesenchymal stem cells by downregulating CCAAT/enhancer-binding protein δ expression through the inhibition of JNK 115. Other extracts of Artemisiae Annuae Herba have been shown to inhibit the expression of a target of PPARγ, the gene encoding fatty acid-binding protein 4, in adipocytes 116. In a study using a high-fat diet-induced rat model of obesity, Artemisiae Annuae Herba extracts notably reduced body weight, fat accumulation, adipocyte size, and serum levels of total cholesterol and triglycerides 114,117. These findings suggest that Artemisiae Annuae Herba components could be effective in preventing and treating obesity and associated metabolic disorders.

**Anti-osteoporotic:** Several pieces of experimental evidence have suggested that additional exploration of roles for Artemisiae Annuae Herba in treatment of osteoporosis is warranted. For example, osteoporosis was prevented by an Artemisiae Annuae Herba extract in a study in ovariectomized mice, in which estrogen deficiency typically causes osteoporosis 118. Additional research has indicated that Artemisiae Annuae Herba and its associated components 114, artemisinin and artemisinin B, exhibit anti-osteoporotic activity by downregulating the activity of the transcription factors c-Fos and nuclear factor of activated T cells 1 (NFATC1), which in turn inhibit osteoclast differentiation as induced by receptor activator of nuclear factor-κB ligand (RANKL) 120,121.

Beyond these diverse clinical activities, Artemisiae Annuae Herba components exhibit complex yet promising anti-cancer activities 122. A deeper understanding how these agents benefit human health in multiple contexts promises to provide intriguing insights at the micro and macro levels into their potential use in cancer therapy.

## From Molecular Structure to Mechanisms: A Forward Reasoning Approach

**Figure 3:**
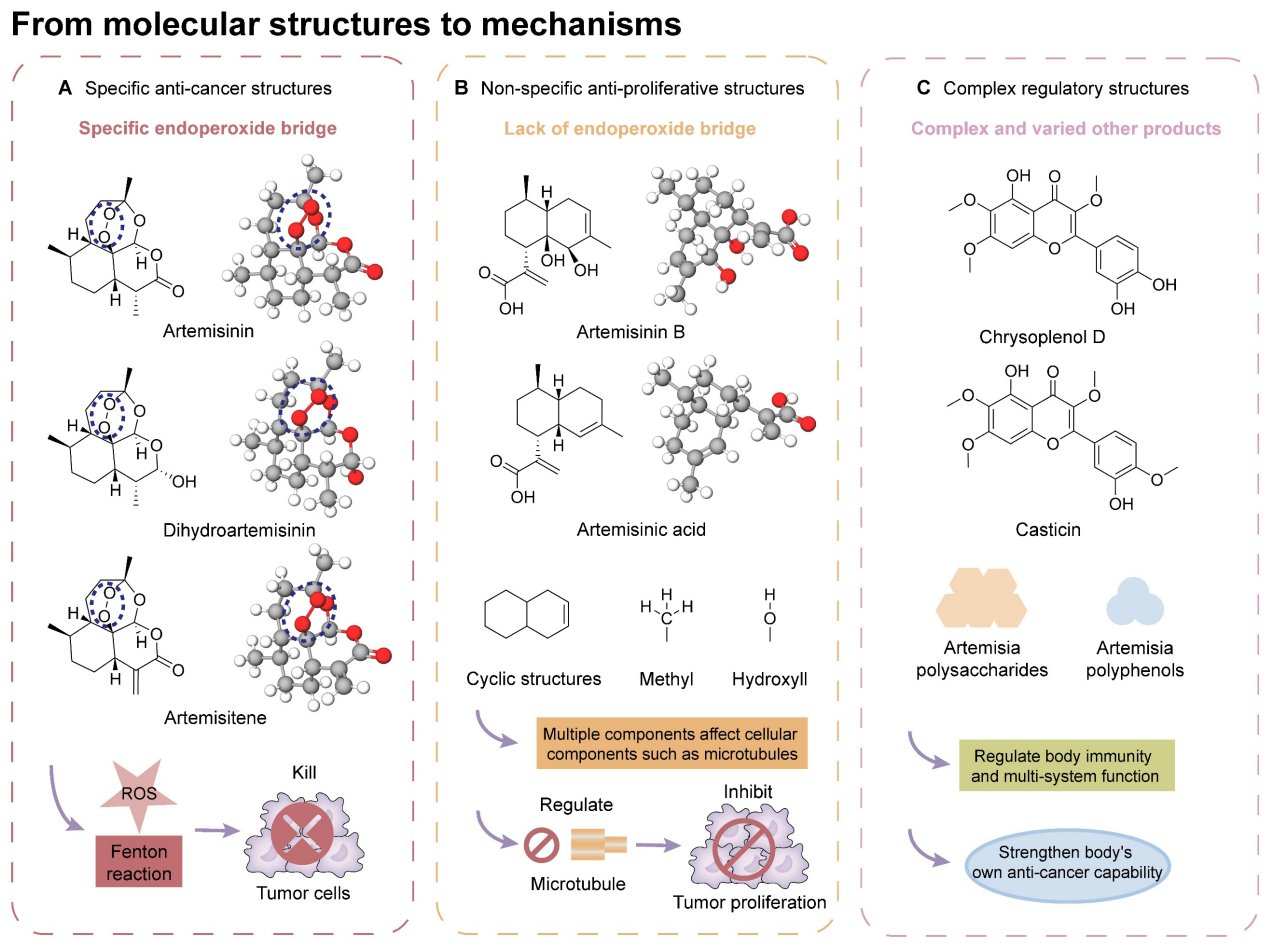
** From molecular structures to mechanisms:** Possible anti-cancer mechanisms have been found upon structural analyses of various molecular components of Artemisiae Annuae Herba, and three possible exploration directions have emerged: specific anti-cancer structures, non-specific anti-proliferative structures, and complex regulatory structures. **A)** Specific anti-cancer structures rely on their unique peroxide bridge, which generates reactive oxygen species under certain conditions to kill tumor cells. **B)** Non-specific anti-proliferative structures are associated with the cyclic structures and certain functional groups of sesquiterpenoids, influencing cell proliferation through various mechanisms. **C)** Complex regulatory structures primarily involve diverse polysaccharides and polyphenols, which modulate immune responses and improve the body's anti-cancer capabilities.

Traditional Artemisiae Annuae Herba-based medications contain a multitude of components 123, including a series of sesquiterpenes, coumarins, lignans, phloroglucinol derivatives, and numerous polysaccharides and polypeptides, all of which can be isolated and purified 124. Among these, certain unique constituents of Artemisiae Annuae Herba exhibit distinct biological properties and medicinal value. In addition, each component seems to work through distinct anti-cancer mechanisms. To further elucidate the possible associations and interactions between these components and tumor tissues, a micro-level analysis of Artemisiae Annuae Herba components and their foundational mechanisms serves as an insightful focus and exploratory direction *(Figure 3)*. This approach could catalyze a substantial paradigm shift in Artemisiae Annuae Herba research.

Extensive structural analyses of Artemisiae Annuae Herba components have facilitated comparisons of the biological characteristics and bioactive mechanisms of its various components. Furthermore, inter-group comparisons and analyses have been conducted to identify similarities and differences between Artemisiae Annuae Herba components and anti-cancer drugs derived from other traditional herbal medicine and natural plants 120. Overall, the potential anti-cancer capabilities of Artemisiae Annuae Herba, in terms of molecular structure and micro-foundational aspects, can be broadly categorized into the following three areas: specific anti-cancer structures, non-specific anti-proliferative structures, and structures that interact with complex regulatory components.

### Chemical structures that specifically target cancer

Considering its potent anti-malarial properties and minimal side effects 126, a central question that has intrigued scholars is whether artemisinin and its derivatives can exhibit similar specificity against various proliferative tumors while minimizing harm to the body 127. The exceptional anti-malarial effectiveness of artemisinin and its derivatives is primarily due to their unique “endoperoxide bridge” structure, a specific chemical bond between oxygen atoms in the ring, distinguishing them from other natural plant components and sesquiterpene compounds 128. During the red blood cell stage of the *Plasmodium* lifecycle, the malaria parasite ingests and digests hemoglobin, releasing a substantial amount of reductive heme and ferrous ions. These reductive components serve as the basis for artemisinin's specific parasiticidal action. Catalyzed by divalent iron, the endoperoxide bridge opens, generating free radicals that target and disrupt proteins, lipids, and DNA 124,125. This suggests that if artemisinin-related compounds possess a strong anti-cancer effect, the molecular mechanism is likely rooted in this distinctive endoperoxide bridge 38,126,127.

It is important to note, however, that the potential for this molecular structure to confer specific anti-cancer activity remains a topic of considerable debate 128,129. The endoperoxide bridge and its related Fenton reaction, known for producing alkyl radicals and ROS, are well-documented 130, though their role in anti-cancer processes remains controversial 131-134. Nan *et al.* demonstrated a direct link between the endoperoxide bridge of artemisinin and its derivatives and its anti-cancer action in the MCF-7 breast cancer cell line 135. They observed that artemisinin, which contains the endoperoxide bridge, exhibited significantly stronger cytotoxicity compared to deoxyartemisinin, which lacks this structure. Nevertheless, many researchers and mainstream opinions remain skeptical of such specific anti-cancer effects 141,142. Given the complex pathology and diverse microenvironments of various tumors, even though tumors are often rich in hemoglobin and transferrin due to extensive angiogenesis, they do not universally provide the ferrous ions and hemes necessary to support a Fenton reaction akin to malaria infection. Therefore, the potential anti-cancer mechanism established by the endoperoxide bridge structure may only be effective in patients with tumors rich in ferrous ions.

### Chemical structures with non-specific anti-proliferative effects

In addition to artemisinin, structural analyses of other Artemisiae Annuae Herba components have also prompted new considerations. Artemisinic acid, a precursor to artemisinin, lacks the peroxide bridge, yet some studies have demonstrated its anti-cancer properties 138,139. This finding suggests that the unique peroxide bridge of artemisinin is not the only aspect of Artemisiae Annuae Herba components that is endowed with anti-cancer potential. The most abundant components of Artemisiae Annuae Herba, terpenoids, are also noteworthy in existing pharmaceutical research 140. In general, terpenoids are prevalent plant-derived natural products and include well-known chemotherapeutic agents such as paclitaxel, as well as several anti-cancer drugs have been refined from TCM components, such as elemene. The sesquiterpenoids in Artemisiae Annuae Herba share similarities with these recognized plant-based anti-cancer agents, presenting an intriguing avenue for exploration.

It is noteworthy that while the molecular foundations for the anti-cancer activities of paclitaxel, elemene, and other terpenoids differ, their mechanisms of action exhibit some similarities 141. Specifically, multiple studies have suggested that the anti-cancer mechanisms of plant terpenoids are linked to varying degrees with interruption of the cell cycle and of tubulin microtubule polymerization 142-145. For example, paclitaxel's anti-cancer efficacy primarily relies on its tricyclic structure, which facilitates binding to tubulin 146; the two benzene rings in its tricyclic structure closely interact with the hydrophobic regions of microtubule proteins, leading to the formation of stable complexes 152,153. Additionally, the acetyl group on carbon-10 of paclitaxel forms hydrogen bonds with tubulin, collectively inhibiting microtubule polymerization and cell cycle proliferation 149. The mechanism underlying elemene's anti-cancer effect is more complex, with numerous studies indicating its association with cell cycle arrest 155,156. The predominant theory suggests that elemene inhibits tubulin polymerization by inhibiting the MAPK pathway 152,153.

Research on several components of Artemisiae Annuae Herba has also suggested the existence of such mechanisms. For example, Wu *et al.* showed that DHA altered activity of the p38/MAPK signaling pathway 154, and in colitis-associated colorectal cancer, DHA has been shown to suppress phosphorylation associated with the p38/MAPK pathway, leading to cell cycle arrest 155,156. While no direct evidence has shown that Artemisiae Annuae Herba components share structural similarities with plant-based anti-cancer agents that target tubulin directly, the influence of Artemisiae Annuae Herba components on intracellular signaling pathways 157-159, including MAPK/PI3K/Akt, highlights their role in cytoskeleton dynamics and cell cycle arrest 160-162. These structures that exert nonspecific anti-proliferative actions may significantly contribute to the efficacy of Artemisiae Annuae Herba components against rapidly proliferating tumor cells.

Certainly, regulating signaling pathways, influencing intermolecular interactions of tubulin, inhibiting microtubule polymerization, and arresting the cell cycle would not have effects that are specific to tumor cells. More accurately, they mirror the nonspecific anti-proliferative effects of alkylating agents in traditional chemotherapy, targeting all rapidly proliferating tissues 168, with cancerous tissues being the most affected. Accordingly, if the biochemical basis of the anti-cancer activity of Artemisiae Annuae Herba components leans more towards this anti-proliferative capability, the dosage of these drugs becomes a critical concern, and associated side effects can no longer be overlooked 137,157,164,165. Future research should aim to determine which Artemisiae Annuae Herba components exhibit optimal anti-proliferative effects with enhanced drug efficacy and minimized side effects.

### Chemical structures with complex effects on regulatory mechanisms

In broadening the scope of components related to Artemisiae Annuae Herba, it is crucial to consider not only the structurally similar sesquiterpene compounds with relatively high molecular weights but also the diverse array of other components that play significant roles in the foundational conditions for anti-cancer activity 166. Among these varied components, certain polysaccharides can enhance anti-cancer effects through their unique immune-related properties 11,167, which are rooted in their structural characteristics. For instance, Chen *et al.* demonstrated that *Artemisia* polysaccharides inhibit the growth of liver cancer tumors in mice 172. This effect was primarily attributed to the distinctive molecular structure of these polysaccharides, which stimulates and enhances the antigen recognition capabilities of lymphocytes in the mouse model, thereby promoting the destruction of liver cancer cells through both cellular and humoral immune mechanisms 168.

The complex composition and lack of specific pharmacological properties in some secondary components, such as polysaccharides and polypeptides, prevent them from becoming the primary foundation of Artemisiae Annuae Herba's anti-cancer capabilities. Nevertheless, their presence underscores the significance of Artemisiae Annuae Herba as a TCM with indispensable auxiliary components. Research into the relationship between these components and anti-cancer activity holds promising potential for developing adjunctive anti-cancer regulatory mechanisms 169.

Overall, components related to Artemisiae Annuae Herba indeed possess micro-molecular structural foundations that strongly influence their anti-cancer mechanisms. These foundations include the specific endoperoxide bridge structures of artemisinin and its derivatives, non-specific cell cycle-related anti-proliferative structures, and various regulatory synergistic effects. The existence of these differential foundations further establishes the comprehensiveness of the anti-cancer activities of Artemisiae Annuae Herba components, highlighting the potential application value of this herb in the advancement of cancer treatment.

## From Efficacy to Mechanisms: A Reverse Reflection on Anti-Cancer Actions

A structural analysis of the molecules that make up Artemisiae Annuae Herba preparations suggests that the herb exhibits a comprehensive and multidirectional anti-cancer potential. This potential can be attributed to the complex composition of the formulations and to its varied interactions with biological components. Complementing this approach, a reverse efficacy analysis further supports the herb's potential as an anti-cancer agent. This potential has been rigorously validated through ongoing research and repeated experimental studies. Artemisinin and its derivatives, including artesunate and DHA, have been studied for their anti-cancer activities since the late 1990s 43,143,175,176. These compounds have been shown to promote apoptosis of cancer cells 172-174, inhibit tumor angiogenesis 15,56,180, and block tumor invasion and metastasis 158,176.

Extracts of Artemisiae Annuae Herba, in addition to purified artemisinin, also exhibit significant anti-cancer activity and tumor-killing effects via multiple regulatory mechanisms, as supported by extensive *in vitro*, *in vivo*, and clinical research 127,182. For instance, Michaelsen *et al.* reported a clinical study involving patients with advanced prostate cancer, where the long-term addition of Artemisiae Annuae Herba following short-term treatment with bicalutamide led to tumor regression and treatment remission, as confirmed by prostate-specific antigen (PSA), magnetic resonance imaging, and SPECT/CT indicators 122. Furthermore, a wide range of natural components that have been isolated from Artemisiae Annuae Herba, including polysaccharides and polyphenols, have garnered significant attention for their potential as anti-cancer drugs 178-181.

**Figure 4:**
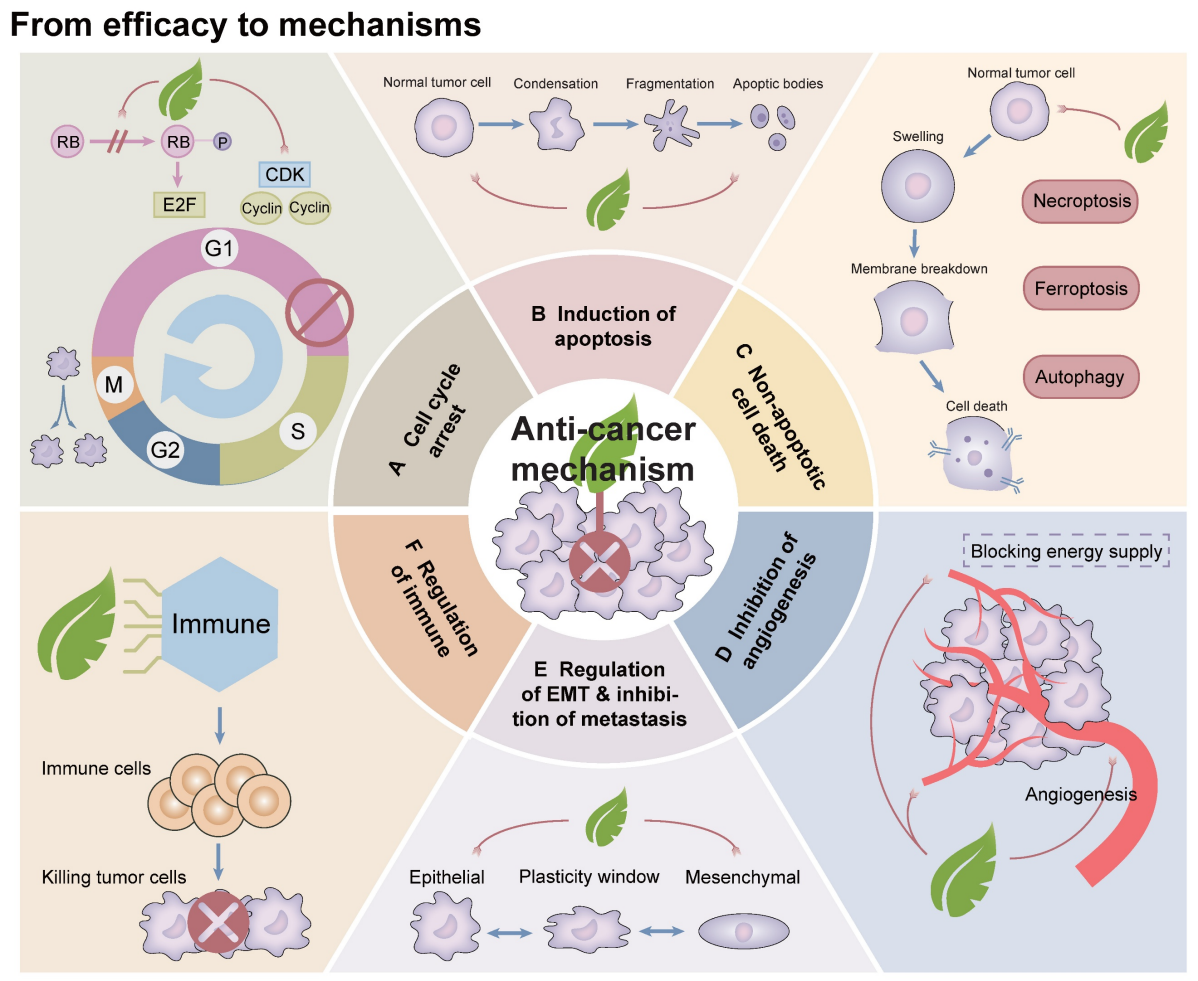
** From efficacy to mechanisms:** Experiments investigating the anti-cancer activities of existing Artemisiae Annuae Herba components were analyzed and summarized, and possible anti-cancer mechanisms were deduced. Improvements to experimental strategies were proposed. At present, the mechanisms can be summarized into six aspects: cell cycle arrest, induction of apoptosis, induction of non-apoptotic cell death, inhibition of angiogenesis, regulation of EMT and inhibition of metastasis, and regulation of immune functions.

After reviewing and synthesizing numerous studies on Artemisiae Annuae Herba components, we found that the primary anti-cancer mechanisms can be categorized into six key functions *(Figure 4)*: (1) induction of cell cycle arrest; (2) induction of apoptosis; (3) induction of non-apoptotic cell death processes, including autophagy, ferroptosis, pyroptosis, and macrophage death; (4) inhibition of angiogenesis; (5) regulation of epithelial-mesenchymal transition (EMT); and 6) modulation of immune system functions. This analysis revealed unique primary effects and synergistic interactions of Artemisiae Annuae Herba components, collectively enhancing the plant's notable anti-cancer properties *(Table 1)*.

### Induction of cell cycle arrest

Uncontrolled and exceptionally rapid cell division is a fundamental characteristic of tumor cells, driving their proliferation and contributing to disease progression 182. The process of cell division, known as the cell cycle, consists of four major phases: G1, S, G2, and M phases. This cycle is tightly regulated by cyclins and cyclin-dependent kinases (CDKs), which play critical roles in ensuring orderly progression through each phase 188. Recent studies have identified artemisinin and its derivatives as potential modulators of cyclins and CDKs, highlighting their promise in the development of cancer therapies 184,185. For example, research on prostate cancer cells has demonstrated that artemisinin inhibits the action of the cell cycle-regulatory protein pRb by disrupting a complex formed among pRb, E2F transcription factors, and CDKs 122. This activity halts the transition of cells from the G1 to the S phase, thereby inhibiting cell cycle progression and suppressing cancer cell proliferation 191. Similarly, in a study on non-small cell lung cancer, Rassias *et al.* found that an extract of dried leaves from Artemisia annua induced G2/M phase and mitotic arrest in PC9 and H1299 cell lines, while also causing G1 phase arrest in A549 cells 53. Collectively, these findings underscore the anti-cancer potential of Artemisiae Annuae Herba components, particularly through their ability to regulate the cell cycle and impede cancer progression 187,188.

### Induction of apoptosis

**Figure 5:**
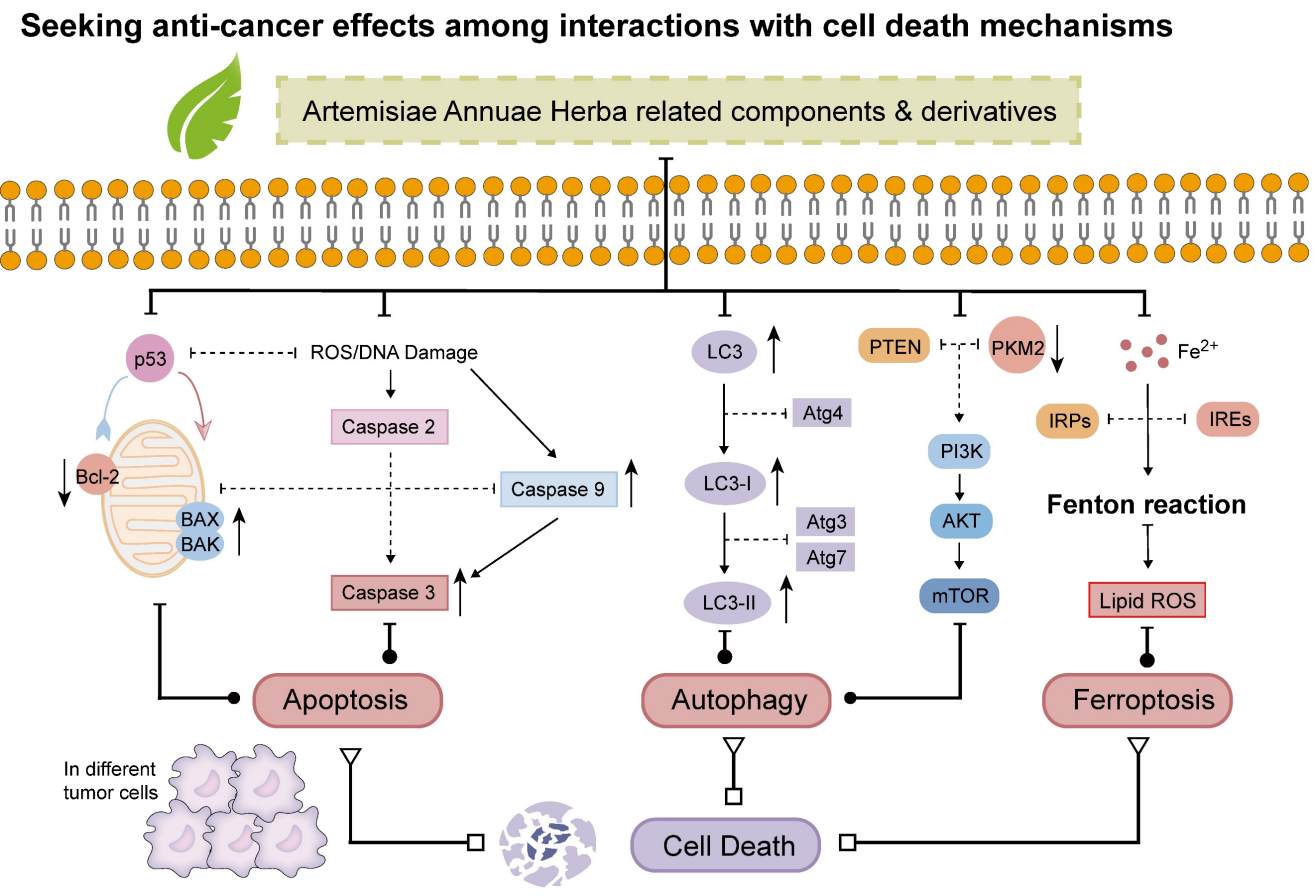
** Seeking anti-cancer effects among interactions with cell death mechanisms:** Artemisiae Annuae Herba components and derivatives can often exert their anti-cancer effects through multiple mechanisms that induce tumor cell death. These mechanisms include **A)** apoptosis, potentially through activation of caspase pathways (through caspase-3 and -9) or modulation of Bcl-2 family proteins, specifically Bcl-2, BAX and BAK; **B)** autophagy, influenced by IL-3 and the PI3K/AKT/mTOR pathway; and **C)** ferroptosis, which is associated with labile iron and the Fenton reaction.

Apoptosis, a form of programmed cell death, is regulated by a complex interplay of various apoptosis-related proteins 189. This process involves two main pathways: the exogenous (death receptor) pathway and the endogenous (mitochondrial) pathway 190. The Bcl-2 family proteins play a central role in both of these mechanisms, encompassing pro-apoptotic members such as Bcl-2-associated X protein (Bax), Bcl-2 homologous antagonist killer protein, and Bcl-2-associated death promoter, as well as anti-apoptotic proteins like Bcl-2 itself 191,192. These proteins are tightly regulated by the tumor suppressor protein p53, which is critical in mediating programmed cell death, particularly in cancer cells under the influence of therapeutic agents 193,194. Interactions with the apoptotic cell death machinery underlie one of the most important anti-cancer mechanisms of Artemisiae Annuae Herba components* (Figure 5)*.

In colorectal cancer cell lines, artemisinin and its derivatives, the components of Artemisiae Annuae Herba, have been shown to induce apoptosis by activating Bax 195. This activation leads to the release of cytochrome C, a key event in the intrinsic apoptotic pathway, ultimately resulting in cell death and an anti-cancer effect 196,197. Additionally, the caspase-dependent pathway, another important mechanism of endogenous apoptosis, has garnered attention in anti-cancer research involving artemisinin and its derivatives 198-200. For instance, in human gastric cancer cell lines, artesunate has been found to promote apoptosis through the activation of caspases-3 and -9, leading to tumor cell death 206. Similarly, Lu *et al.* demonstrated that DHA activates caspase-3 in human lung adenocarcinoma cells (ASTC-a-1), thereby inducing apoptosis and exhibiting significant anti-cancer effects 207,208.

Beyond artemisinin and its derivatives, chrysosplenol D, a flavonol isolated from Artemisiae Annuae Herba, has also shown notable anti-cancer activity, particularly in oral squamous cell carcinoma 204. The results of *in vitro* studies have indicated that PI3K/AKT, extracellular signal-regulated kinase, c-Jun N-terminal kinase, and p38 mitogen-activated protein kinase are downregulated by chrysosplenol D. This inhibition synergizes with the induction of poly(ADP-ribose) polymerase by chrysosplenol D. Additionally, chrysosplenol D further upregulates heme oxygenase-1, leading to apoptosis. Both casticin and chrysosplenol D from Artemisiae Annuae Herba have been shown to induce apoptosis in another way by inhibiting topoisomerase IIα in human non-small-cell lung cancer cells 180. Taken together, these findings underscore the idea that the induction and regulation of apoptosis constitute a pivotal mechanism underlying the anti-cancer effects of Artemisiae Annuae Herba components.

### Induction of non-apoptotic cell death

In addition to the programmed cell death mechanism apoptosis, various forms of non-apoptotic cell death 205,206, such as autophagy 212 and ferroptosis 208, have emerged as critical targets in contemporary cancer therapies 209. Consequently, whether Artemisiae Annuae Herba components induce tumor cell death through these non-apoptotic pathways has garnered significant attention* (Figure 5)*.

Evidence suggests a connection between Artemisiae Annuae Herba components and the induction of autophagy in tumor cells 165. For example, Hsieh *et al.* demonstrated that chrysosplenol D enhances autophagy in oral squamous cell carcinoma cells 204. Specifically, cells treated with chrysosplenol D exhibited an increased accumulation of microtubule-associated protein 1A/1B-light chain 3 within autophagosomes and autophagolysosomes, alongside enhanced autophagosome formation. Similarly, Son *et al.* reported the effects of MC-4, a partially purified artemisinin-based material, in a clinical trial involving patients with metastatic renal cell carcinoma 210. MC-4 was shown to enhance the expression of phosphatase and tensin homolog (PTEN), subsequently downregulating the downstream effector Akt/pyruvate kinase muscle isozyme M2 (PKM2). This reduction in PKM2 activity decreased glucose transporter 1 expression, effectively disrupting cancer cell metabolism. Ultimately, these effects promoted autophagy-related cell death through the regulation of the PI3K/Akt/PKM2 and mTORC1 pathways. Meanwhile, in breast cancer research, ART has been found to upregulate the expression of beclin 1, an autophagy initiator, ultimately exerting anti-cancer effects through downstream cascade reactions 211.

Ferroptosis, an iron-dependent form of cell death driven by the accumulation of lipid peroxides on cell membranes, has emerged as a promising therapeutic strategy for various diseases, particularly cancer 217-219. Artemisiae Annuae Herba components, including artemisinin and its derivatives such as DHA, artesunate, and artemether, have demonstrated the ability to upregulate free iron levels in cancer cells and promote the accumulation of intracellular lipid peroxides 174,220. This dual action induces ferroptosis in cancer cells, thereby inhibiting tumor progression. Notably, these compounds hold potential not only as direct inducers of ferroptosis but also as adjuncts that enhance the efficacy of other cancer therapies 221. For example, Chen *et al.* showed that DHA induces lysosomal degradation of ferritin, a process distinct from traditional autophagy, thereby increasing intracellular free iron and sensitizing cells to ferroptosis 142. Additionally, DHA interacts with intracellular free iron to stimulate iron regulatory proteins (IRPs), which bind to mRNA molecules containing iron-responsive elements (IREs). This interaction modulates the IRP/IRE system, further disrupting iron homeostasis and elevating free iron levels. Furthermore, DHA has been shown to amplify ferroptosis in cancer cells with high tolerance to this form of cell death, particularly by enhancing the effects of glutathione peroxidase 4 inhibition, as demonstrated *in vitro* and in mouse models 14,215. Collectively, these findings suggest that Artemisiae Annuae Herba components sensitize tumor cells to ferroptosis by intricately regulating cellular iron homeostasis.

### Inhibition of tumor angiogenesis

During tumor growth and proliferation, the tumor requires a substantial supply of nutrients and energy to sustain its development 217,218. Solid tumors disrupt the balance between pro-angiogenic and anti-angiogenic factors 219,220, leading to the upregulation of angiogenic stimulators such as matrix metalloproteinases (MMPs) and vascular endothelial growth factor (VEGF), while simultaneously suppressing angiogenesis inhibitors like thrombospondin and tissue inhibitors of metalloproteinases (TIMP) 226. This imbalance facilitates the enhanced blood supply necessary to support the unrestricted growth of tumor cells 227. Furthermore, under hypoxic and nutrient-deficient conditions, tumor cells activate transcription factors such as hypoxia-inducible factor-1α (HIF-1α) and NF-κB, which further drive the expression of VEGF to promote angiogenesis 223,224.

Building on this understanding of tumor vascular regulation, studies have shown that Artemisiae Annuae Herba components exhibit anti-angiogenic properties 72,230. For instance, in mouse embryonic stem cells, artemisinin was found to reduce the levels of HIF-1α and VEGF 226. Wang *et al.* showed that oral administration of artemisinin significantly suppressed tumor angiogenesis in a C57BL/6 mouse Lewis lung cancer model by downregulating VEGF-C expression 227. Similarly, Chen *et al.* demonstrated that artesunate inhibited tumor angiogenesis in BALB/c nude mice implanted with human ovarian cancer cells 228. This inhibition was achieved by reducing the expression of VEGF and its receptor KDR/flk-1, ultimately leading to the suppression of tumor growth.

### Regulation of EMT and suppression of metastasis

The invasion and metastasis of malignant tumors typically begin with the detachment of tumor cells and the reorganization and degradation of the extracellular matrix (ECM) 234-236. This process enables tumor cells to spread and colonize other parts of the body through direct extension, blood circulation, lymphatic pathways, and other mechanisms 237,238. Tumor cell detachment is strongly associated with the downregulation of E-cadherin 239,240 and the degradation of ECM components by proteases such as MMPs 241, both of which play pivotal roles in tumor migration, invasion, and metastasis. Thus, interventions targeting these molecular mechanisms have the potential to impede tumor progression and enhance the efficacy of cancer therapies.

The impact of Artemisiae Annuae Herba components on these targets has garnered significant attention 161. Artemisinin has been shown to markedly reduce MMP2 levels, thereby preventing tumor cell migration in human melanoma cells 242. DHA inhibits cell migration and metastasis by suppressing NF-κB activity and reducing MMP2 and/or MMP9 expression in human breast cancer cells 243 and pancreatic cancer cells 239. Similarly, in non-small cell lung cancer cells, artemisinin was found to inhibit metastasis by downregulating MMP activity and NF-κB signaling 240,241.

Further evidence has highlighted artemisinin's role in modulating E-cadherin expression and ECM stability 242,243. In hepatocellular carcinoma cells, artemisinin significantly upregulated E-cadherin expression while downregulating MMP2 and TIMP-2 levels, contributing to ECM stabilization 158. Similarly, in human colorectal cancer cell lines, artemisinin enhanced E-cadherin expression, altered β-catenin subcellular localization, and suppressed the activity of the Wnt signaling pathway 244. These effects collectively induced apoptosis and inhibited tumor migration and metastasis.

### Modulation of the immune system

Both specific and non-specific immunotherapies play important roles in current cancer treatment strategies 245,246. Specific immunotherapies, such as immune checkpoint inhibitors, CAR T-cell therapy, and tumor vaccines, are advancing rapidly and are often hailed as potential solutions to many cancer-related challenges 252. However, non-specific immunotherapy also remains an indispensable component of cancer treatment 248,249. This approach leverages the innate immune system to target and eliminate tumor cells by activating non-specific immune cells through immunomodulators, thereby promoting the proliferation of immune-active substances and lymphocytes to exert anti-cancer effects 255.

Regarding Artemisiae Annuae Herba, previous studies have predominantly highlighted its role as an immunosuppressant 251,252. This is largely attributed to the anti-inflammatory properties of artemisinin, which enhance its efficacy in treating autoimmune and allergic diseases 253-255. Nevertheless, the broad-spectrum anti-parasitic, anti-bacterial, and anti-fungal activities of Artemisiae Annuae Herba have led researchers to recognize its complex and multifaceted immunoregulatory effects 261. Notably, polysaccharides AAP-1, AAP-2, and AAP-3 extracted from Artemisiae Annuae Herba have demonstrated significant immunomodulatory activities in mouse macrophage experiments. These studies revealed a positive correlation between the levels of interleukin-6 and tumor necrosis factor-α and the supplementation of AAP *in-vitro*, indicating the potent immunostimulatory activity of these polysaccharides 11,171. Further investigation into the underlying mechanisms revealed that AAPs from Artemisiae Annuae Herba extracts can be recognized by TLRs, leading to macrophage activation and the subsequent release of immune-related cytokines.

Drawing from extensive scientific literature and clinical guidelines, immunoregulatory therapies have consistently maintained a significant role in cancer treatment 262. The polysaccharide components isolated and purified from Artemisiae Annuae Herba have shown potential as immune function regulators, paving the way for the development of anti-cancer agents.

### Duality and unity of Artemisiae Annuae Herba in cancer therapy

Clearly, the anti-cancer effects of Artemisiae Annuae Herba extend beyond merely targeting a specific gene or signaling pathway. Instead, its action represents a multidirectional and comprehensive macro-control approach against cancer. The efficacy of Artemisiae Annuae Herba in treating tumors is influenced by the organismal status of various cancer types and the composition of different drug groups, which determine the dominant components and effective mechanisms at play 13. Notably, the anti-cancer potential of Artemisiae Annuae Herba is primarily driven by cell death induced through the tumor cytotoxicity of Artemisiae Annuae Herba components 119,258. Additionally, the modest immunosuppressive effects of artemisinin, along with the complementary role of polysaccharides in non-specific immune activation, further enhance its anti-cancer capabilities 168. The anti-cancer efficacy of Artemisiae Annuae Herba is thus elevated through the favorable interaction and synergistic unity among its various components.

## Feasibility of Using Artemisiae Annuae Herba in Cancer Treatment

**Figure 6:**
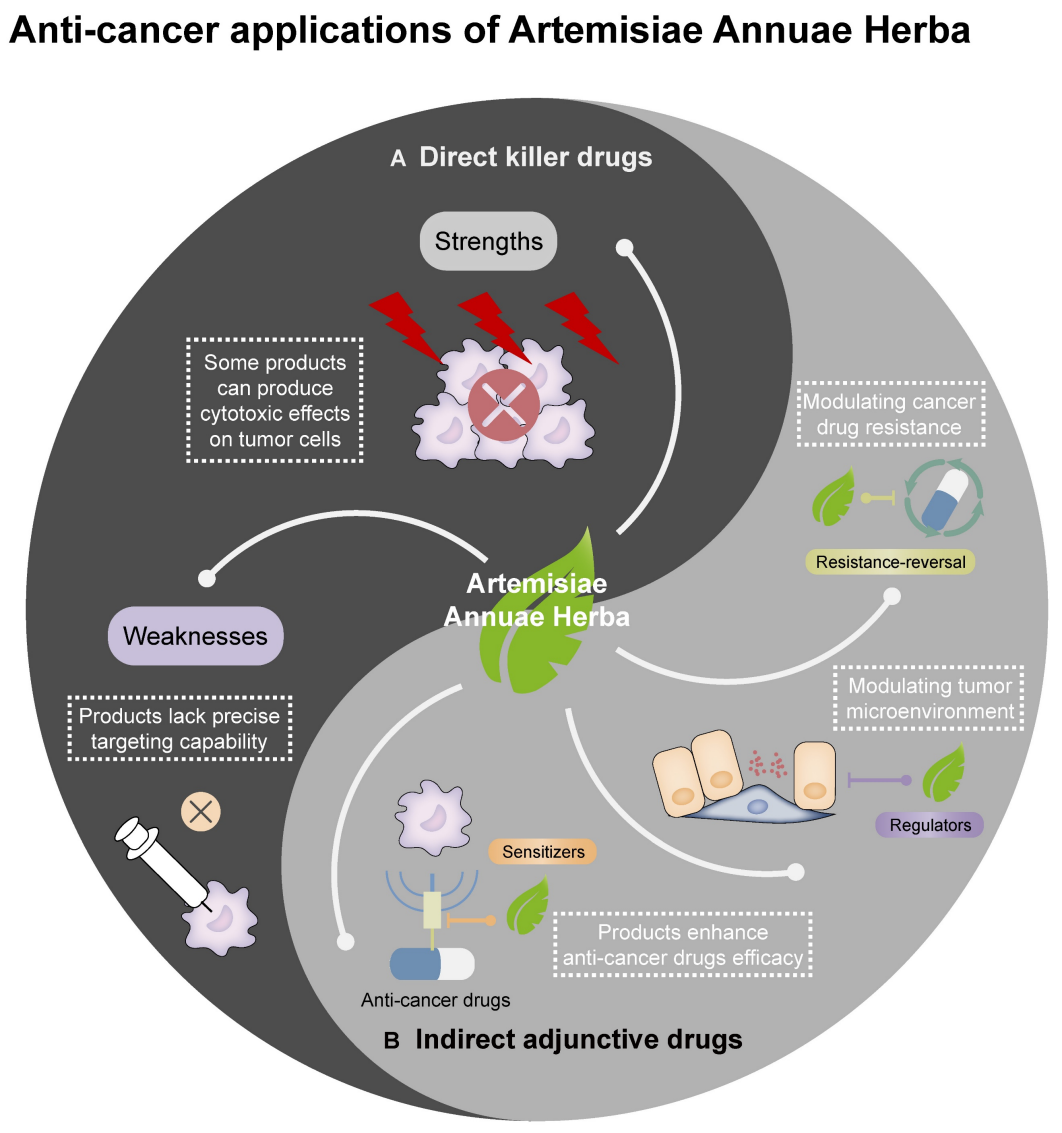
** Anti-cancer applications of Artemisiae Annuae Herba:** The traditional Chinese medicine Artemisiae Annuae Herba may be utilized in cancer therapies as direct killer drugs and as indirect adjunctive drugs in connection with the application of other Chinese medicines.

Artemisiae Annuae Herba exhibits diverse and rational anti-cancer mechanisms, with significant anti-cancer effects observed in numerous experiments both *in vitro* and *in vivo*. However, in recent years, there has been a noticeable lack of breakthroughs in clinical research related to the anti-cancer effects of this preparation 13, representing a critical gap that poses challenges for the use of Artemisiae Annuae Herba to address cancer by the medical community. This issue is largely attributable to the entrenched perception of artemisinin as primarily an anti-malarial agent. Both clinicians and cancer patients often fail to associate Artemisiae Annuae Herba with its potential anti-cancer applications and may even dismiss such claims. This cognitive bias has hindered the exploration and development of Artemisiae Annuae Herba as an anti-cancer agent. In this regard, it is essential to recognize that Artemisiae Annuae Herba components encompass more than a few isolated derivatives 264,265; the plant contains numerous bioactive components with potential therapeutic benefits. Therefore, a comprehensive feasibility assessment of artemisinin and other complex components of Artemisiae Annuae Herba as candidates for cancer therapies remains a critical area for further investigation *(Figure 6)*.

### Comparisons of the anti-cancer activities of Artemisiae Annuae Herba components and existing drugs

Feasibility analyses and evaluations of Artemisiae Annuae Herba components as anti-cancer agents can be initiated by comparing them with conventional anti-cancer drugs approved by the FDA. Current anti-cancer combination therapies primarily include chemotherapy drugs, molecular targeted drugs, immunotherapy drugs, and endocrine therapy drugs, among other less conventional drugs. Due to the complex composition of Artemisiae Annuae Herba components, it is challenging to categorize all of the components under any single existing drug class; instead, artemisinin and other components often below within multiple categories.

The primary components of Artemisiae Annuae Herba are sesquiterpenes, which exhibit structural similarities to anti-cancer agents derived from other medicinal plants 261,262. As discussed above, structural analyses have revealed that Artemisiae Annuae Herba mediates cytotoxic effects and proliferation inhibition via the distinctive peroxide bridge structure of artemisinin and via other non-specific structural interactions 169,225,263. However, compared to current alkylating agents and chemotherapeutics (e.g., paclitaxel and cisplatin), Artemisiae Annuae Herba exhibits weaker cytotoxicity against tumor cells. Nevertheless, the peroxide bridge structure and iron-dependent Fenton reaction mechanisms merit rigorous investigation to validate their therapeutic feasibility and efficacy.

In advancing the herb's anti-cancer applications, the combination of Artemisiae Annuae Herba components with transferrin-mediated drug delivery systems has emerged as a particularly promising strategy 269-271. This approach leverages both the intrinsic anti-cancer properties of Artemisiae Annuae Herba and the tumor-targeting capabilities of transferrin receptors (TfR1), which are overexpressed in many malignancies. Transferrin, a plasma glycoprotein responsible for iron transport, binds to TfR1 with high affinity. Since TfR1 is overexpressed in many cancers, including breast 267,268, lung 269,270, and pancreatic 271,272, transferrin has been widely explored as a “Trojan horse” for targeted drug delivery. By conjugating artemisinin derivatives to transferrin or encapsulating them within transferrin-coated nanoparticles, researchers aim to enhance tumor-specific accumulation while minimizing off-target effects. For instance, a 2022 study demonstrated that transferrin-modified liposomes loaded with DHA achieved higher uptake in TfR1-positive triple-negative breast cancer cells compared to normal cells, significantly improving therapeutic efficacy in models 278. In glioblastoma models, transferrin-conjugated artemisinin nanoparticles penetrated the blood-brain barrier and reduced tumor volume compared to free artemisinin 274.

Leveraging its targeted recognition mechanisms and combination-mediated precision therapy, Artemisiae Annuae Herba differentiates itself from traditional chemotherapy by selectively inducing tumor cell death with minimal systemic toxicity and a favorable safety profile, key advantages for anti-cancer drug development. These safety advantages are supported by clinical studies. For instance, Rassias *et al.* demonstrated that Artemisiae Annuae Herba components effectively suppress tumor growth while exhibiting minimal cytotoxicity toward normal cells, underscoring its translational potential for relatively safe cancer therapies 53. These findings validate Artemisiae Annuae Herba's feasibility as a targeted anti-cancer agent with distinct clinical advantages 122,275.

In contrast to the rapid advancements in immunotherapy, particularly specific therapies like immune checkpoint inhibition and CAR-T therapy, polysaccharides from Artemisiae Annuae Herba primarily exert a non-specific immune regulatory effect akin to thymosin drugs 11,173,281. They influence immune factor-related components within the tumor microenvironment 282. When considering only the immunomodulatory capabilities of Artemisiae Annuae Herba, the isolated and purified components may not exhibit sufficient anti-cancer efficacy. However, its non-specific immune modulation enhances the therapeutic activity of Artemisiae Annuae Herba-derived anti-cancer agents. Moreover, the multi-mechanistic profile can enable Artemisiae Annuae Herba to be developed into a single drug with synergistic component interactions that amplifying its anti-cancer effects. Just as with the specific delivery systems based on transferrin, Artemisiae Annuae Herba-related immunomodulatory preparations are also being further developed. In addition to the synergistic effects on drug resistance and tumor suppression exhibited by combinations of artemisinin with transferrin-based delivery systems 283,284, transferrin-coupled artemisinin formulations have also shown immunomodulatory effects. In a hepatocellular carcinoma study, these nanoparticles promoted M1 macrophage polarization and increased CD8^+^ T-cell infiltration, suggesting potential for multi-mechanism cancer treatment 280.

Beyond comparisons with common drugs, Artemisiae Annuae Herba components share characteristics with certain specialized anti-cancer agents. For instance, some preparations can serve as auxiliary anti-cancer agents with apoptosis inducers like bortezomib 281,282 and can also inhibit tumor neovascularization, akin to angiogenesis inhibitors such as thalidomide 51,283. Additionally, differentiation inducers play a crucial role in treating hematological malignancies 289,290. The potential of artemisinin to function as a differentiation inducer, comparable to all-trans-retinoic acid or arsenical agents, remains underexplored and warrants further research and evaluation 291.

Despite their promising potential, Artemisiae Annuae Herba components exhibit certain weaknesses in their application as anti-cancer agents 127. Unlike the widely used and highly regarded targeted therapies, these preparations lack the capability to specifically target mutated genes or abnormal signaling pathways, which are essential for precise cancer treatment. Consequently, compared to molecularly targeted drugs, Artemisiae Annuae Herba components are less effective in achieving precision-guided tumor cell eradication 127. Their therapeutic specificity for particular tumor mutations is lower, and they cannot minimize individual drug-related side effects to the same extent as molecularly targeted treatments. However, this limitation may also present a unique advantage. While lacking precision, the broad and multifaceted anti-cancer mechanisms of Artemisiae Annuae Herba significantly expand their potential therapeutic applications. Notably, experimental evidence supports the anti-cancer effects of Artemisiae Annuae Herba components in both hematologic malignancies 196,287 and a variety of solid tumors 185,186,293. This versatility underscores their potential utility across a wide range of cancer types. Taking these factors into account, Artemisiae Annuae Herba components hold considerable promise as candidates for future anti-cancer strategies.

### Feasibility of combination therapies that include Artemisiae Annuae Herba components

Cancer therapies often include numerous drugs that do not directly kill tumors but instead work synergistically with other anti-cancer agents 294,295. These drugs, known as sensitizers, are crucial in enhancing the efficacy and reducing the toxicity of treatment regimens 291-293. A significant category of these sensitizers is derived from TCM 294-296.

Among TCM-based sensitizers, Artemisiae Annuae Herba components possess distinctive similarities to certain established compounds and enhance the therapeutic effects of primary drugs by modulating signaling pathways or protein expression 302-304. For instance, in research involving HCT116 colorectal cancer cells, polyphenols isolated from Artemisiae Annuae Herba were found to enhance the anti-cancer effects of β-lapachone against oxaliplatin-resistant strains by inducing DNA damage and regulating apoptosis through multiple mechanisms 179. Similarly, in hematological acute lymphoblastic leukemia, the methanolic extract of Artemisiae Annuae Herba has been shown to potentiate the anti-cancer effects of vincristine by mediating cytotoxicity 263.

Another mechanism by which these drugs operate is by modifying the tumor cell microenvironment, thereby optimizing conditions for the primary anti-cancer drugs 305,306. For example, quercetin, which can be isolated from Artemisiae Annuae Herba, has been shown by Guo *et al.* to regulate the tumor microenvironment in endometrial cancer, inhibiting tumor cell growth and migration 177. However, this study did not further assess the efficacy of Artemisiae Annuae Herba in combination with conventional anti-cancer drugs for endometrial cancer.

Certain drugs can interact with chemotherapy agents or specific structural components of tumor cells through their molecular configurations, enhancing the efficacy of cancer therapies or weakening tumor cell activity to achieve improved therapeutic outcomes 307-309. For instance, Wang *et al.* demonstrated that artemisinin derivatives can bind to rhein to exert dual inhibition on heat shock protein 72 and heat shock cognate 70, thereby enhancing rhein's therapeutic efficacy against liver cancer 305. Similarly, Fu *et al.* revealed that Artemisiae Annuae Herba-derived compounds such as casticin and chrysosplenol D can bind to apical Iα-DNA. This interaction disrupts DNA replication, induces DNA damage, and consequently boosts the effectiveness of anti-cancer drugs in treating non-small-cell lung cancer 180.

Beyond sensitizing agents that enhance the efficacy and mitigate the toxicity of anti-cancer adjuvant drugs, there is growing interest in resistance-reversal agents, which counteract tumor cell drug resistance—a major challenge in current cancer therapies 306-309. To address the frequent emergence of drug resistance among existing anti-cancer agents, research has increasingly turned to TCM for potential solutions 310,311. Notably, Artemisiae Annuae Herba components not only exhibit direct anti-cancer activity across various cancer types but also hold significant potential for reversing drug resistance through multilayered regulatory mechanisms 312-314. For example, Ma *et al.* showed that the artemisinin derivative artesunate can reverse the resistance of hepatocellular carcinoma to sorafenib 315. This effect is achieved by downregulation of expression of actin filament associated protein 1 like 2 protein expression, inhibiting the phosphorylation of inhibiting SRC and FUN14 domain-containing 1, and inducing mitochondrial autophagy and apoptosis in sorafenib-resistant cancer cells. Additionally, flavonoids extracted from Artemisiae Annuae Herba have been shown to regulate P-glycoprotein, a protein closely associated with multidrug resistance in tumor cells 321,322. These findings underscore the potential of Artemisiae Annuae Herba components in overcoming tumor resistance and advancing cancer treatment strategies.

## Challenges in Artemisiae Annuae Herba-Based Cancer Therapy

**Figure 7:**
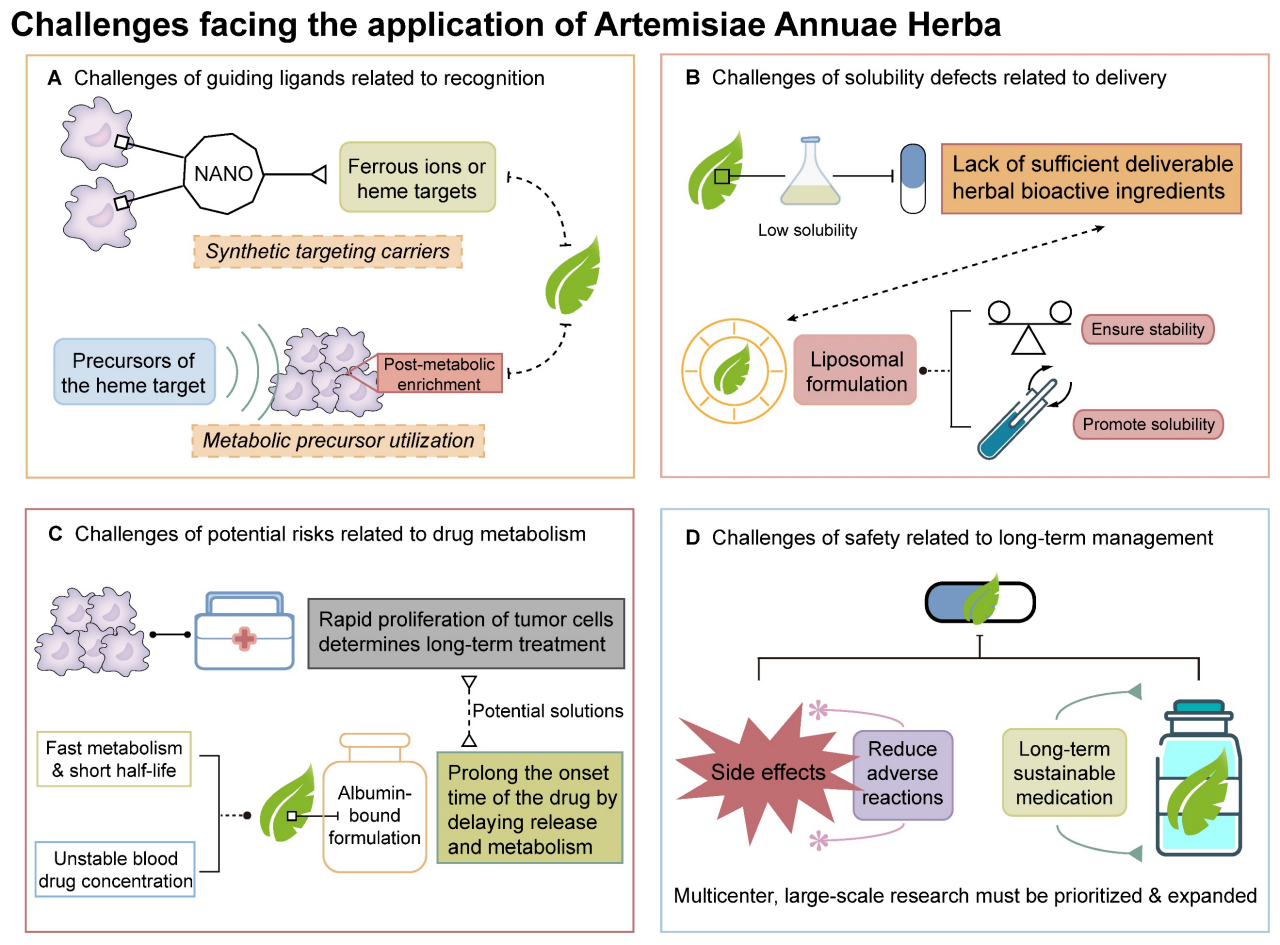
** Challenges facing the application of Artemisiae Annuae Herba:** Although Artemisiae Annuae Herba has bright prospects as cancer treatment, the challenges to its implementation cannot be ignored. The main dilemmas include: **A)** Challenges of guiding compounds to the cancer site: two strategic approaches are proposed, including synthetic targeting carriers and metabolic precursor utilization; **B)** Challenges of solubility and delivery: liposomal formulations are a possible solution; **C)** Challenges of potential risks related to drug metabolism: albumin-bound formulations are proposed as a key strategy; **D)** Challenges of side effects and safety related to long-term management: multicenter large-scale research must be prioritized and expanded.

There are many aspects of Artemisiae Annuae Herba worth discussing in relation to its anti-cancer properties, and its actual efficacy and potential mechanisms have gained significant recognition. However, focusing solely on its positive aspects can lead to undue optimism. It is important to acknowledge that numerous challenges remain before Artemisiae Annuae Herba components can be widely recognized and formally used in cancer treatments *(Figure 7)*.

### Challenges of guiding the components to the target area

While Artemisiae Annuae Herba shows promise as an anti-cancer agent, reducing it to a conventional plant-derived chemotherapeutic would undermine its unique advantages. Similar to its anti-malarial action, the herb's anti-cancer advantages rely on interactions with specific molecular targets, which guide the compounds to the necessary site of action. However, the targets of Artemisiae Annuae Herba components are abundant in the context of malaria, but tumor heterogeneity makes such targets more scarce in the realm of cancer. For example, key components of Artemisiae Annuae Herba, such as artemisinin, artemisitene, and DHA, are known to target heme and ferrous ions 88,323,324. In malaria, heme is abundant due to the parasite's lifecycle, but in tumors, heme/ferrous ion targets are rare owing to genetic diversity and mutations.

To address this challenge, two strategic approaches are proposed. The first approach involves the development of synthetic targeting carriers. These carriers, potentially constructed from nanomaterials, would bind to tumor-specific antigens and locally concentrate heme/ferrous ions, enabling precise tumor targeting by Artemisiae Annuae Herba. Inspired by malaria's heme-targeting mechanism, nanocarriers could be engineered with dual-functional surfaces: one end binds tumor-specific antigens, while the other captures ferrous ions or promotes tumor cells to produce heme. This process would mimic the red blood cell invasion of *Plasmodium*, hijacking tumor cells to amplify drug targeting. The second strategy involves the utilization of heme metabolic precursors. Here, tumor metabolism would be leveraged by administering heme precursors, like aminolevulinic acid, which accumulates in tumors and converts to heme, creating localized targets for Artemisiae Annuae Herba agents 325,326.

Both strategies face significant hurdles: carrier design demands interdisciplinary innovation, while precursor optimization requires extensive trial-and-error validation. Overcoming these challenges is critical to advancing Artemisiae Annuae Herba-based therapies into clinical practice.

### Delivery challenges due to low water solubility

Artemisiae Annuae Herba faces hurdles in anti-cancer applications that are akin to its historical limitations in the context of malaria treatment. Initially, the herb's anti-malarial efficacy was limited by suboptimal extraction methods and the poor aqueous solubility of its active sesquiterpene compounds. The lipophilic nature of sesquiterpenes, the key bioactive components, results in low aqueous solubility, hindering clinical translation 322. Even solubility-enhanced derivatives face stability issues, creating a dilemma between precise dosing requirements for cancer therapy and physicochemical limitations.

Similar solubility challenges plague other plant-derived chemotherapeutics (e.g., paclitaxel and vincristine), yet advanced delivery systems have enabled their clinical success. Innovative drug delivery strategies, particularly liposomal formulations, offer transformative solutions for low-solubility agents. Liposomes enhance stability and enable precise drug loading, mitigating solubility-related bioavailability fluctuations. While Artemisiae Annuae Herba-derived liposomes (e.g., artesunate liposomes) remain experimental, optimizing drug-carrier compatibility, encapsulation efficiency, and payload capacity represent critical research frontiers 323-325.

### Challenges of rapid drug metabolism

In addition to the challenges that are related to those faced for malaria treatment, Artemisiae Annuae Herba agents also face hurdles that are unique to clinical translation for oncology. Unlike malaria, an acute infection, tumor management requires chronic suppression of proliferating cells to maintain a low tumor burden, a distinction rooted in disease pathophysiology. Anti-malarial therapy aims to eradicate parasites in a single course, whereas tumors demand prolonged, cyclical treatment due to their self-renewing, proliferative nature. These prolonged treatments thus require the optimization of dosing regimens for Artemisiae Annuae Herba in oncology.

One potential solution is referred to as short-cycle therapy. Considering the precision anti-cancer action of Artemisiae Annuae Herba components and their association with fewer side effects compared with traditional chemotherapy, it is worth considering shortening dosing intervals during cancer therapy to achieve a shorter total cycle and to allow patients to return to normal life earlier while ensuring the same survival benefit. A second potential solution is to use these agents in maintenance therapy. As long as the risk of significant side effects remains low, it is worth considering long-term administration of drugs derived from Artemisiae Annuae Herba and to no longer routinely discontinue the drug when the tumor burden has decreased. The goal would be to maintain a low-level balance of tumor burden through tumor killing and inhibition of tumor proliferation in order to reduce tumor recurrence and metastasis. Finally, a third possible option is to implement a pulse-dosing strategy. This option is essentially a combination of the other two options in which the agents are delivered at a low level for long-term administration, but the patient receives regular high-dose pulsative shock treatments with appropriate Artemisiae Annuae Herba agents. This strategy could further reduce the occurrence of drug resistance, and we posit that pre-clinical and clinical studies are warranted.

Dosing regimens for Artemisiae Annuae Herba also face pharmacokinetic challenges, particularly in maintaining therapeutic drug concentrations. In malaria therapy, rapid drug clearance of Artemisiae Annuae Herba derivatives can lead to subtherapeutic drug levels, leading to pseudo-resistance. Pharmacokinetic studies have revealed that Artemisiae Annuae Herba components achieve rapid peak plasma concentrations but exhibit short half-lives due to fast metabolism and excretion 326,327. This transient subtherapeutic exposure may be misinterpreted as drug resistance in malaria, complicating treatment outcomes. For anti-cancer applications, these pharmacokinetic limitations pose significant challenges for sustaining therapeutic drug levels. To address this, albumin-bound formulations could prolong the onset time of the drug by delaying drug release and metabolism. Alternatively, developing derivatives with extended half-lives represents a promising research direction.

### Challenges of side effects and safety related to long-term management

Despite the potential of Artemisiae Annuae Herba components to reverse drug resistance in certain tumors, they also exhibit notable resistance issues themselves when employed in cancer therapy. For instance, in a clinical study on Artemisiae Annuae Herba-assisted prostate cancer treatment, researchers observed that although some patients initially experienced tumor control, prolonged oral administration of Artemisiae Annuae Herba capsules was associated with a progressive rise in PSA levels 122. This underscores the necessity for further investigation into the mechanisms of drug resistance and the development of strategies to mitigate this issue.

Anti-cancer efficacy of Artemisiae Annuae Herba components is further complicated by insufficient pharmacotoxicological data on chronic exposure. While acute exposure to artemisinin in anti-malarial therapy has rarely resulted in neurotoxicity 333,334 or anaphylaxis 335, the use of Artemisiae Annuae Herba as a direct anti-cancer chemotherapeutic agent necessitates comprehensive pharmacokinetic and toxicological data, particularly concerning the short-term effects of high doses. When Artemisiae Annuae Herba components are considered for long-term adjuvant cancer therapy, it is imperative to conduct studies on chronic toxicology and drug interactions, especially for patients with chronic diseases requiring prolonged medication. Given these challenges, multicenter, large-scale research on Artemisiae Annuae Herba must be prioritized and expanded.

Despite these challenges, the potential of Artemisiae Annuae Herba components to deliver breakthroughs in cancer treatment is undeniable. The scientific community has amassed a wealth of basic and clinical research data on the anti-cancer effects of Artemisiae Annuae Herba over the past two decades. It is now important to further investigate their clinical application value to benefit cancer patients globally.

## Conclusion and Prospects

The development of new cancer therapies has faced significant bottlenecks, prompting exploration into repurposing natural drugs and TCM agents for cancer treatment. This review examines the potential of Artemisiae Annuae Herba components as anti-cancer agents. It begins by reviewing the historical context of Artemisiae Annuae Herba in both Eastern and Western medicine, highlighting the increasingly sophisticated isolation and purification of its medicinal components, as well as the ongoing redefinition and expansion of its pharmacological effects. The article then elaborates on the diverse and complex, yet interacting, pharmacological effects of Artemisiae Annuae Herba components, including sesquiterpenes, polysaccharides, and peptides, encompassing anti-parasitic, anti-viral, anti-bacterial, anti-fungal, anti-inflammatory, anti-obesity, anti-osteoporosis, and anti-cancer activities. To elucidate the mechanisms underlying its anti-cancer effects, two analytical approaches have been employed. First, a forward reasoning approach from molecular structures to anti-cancer effects posits three independent but synergistic herb structures: the specific peroxy-bridge structures, the non-specific anti-proliferation structures, and the immunomodulatory structures. Second, a reverse reflection from herb response to potential anti-cancer mechanisms details the various modes of action and applications of Artemisiae Annuae Herba components in cancer treatment. These mechanisms include cell cycle arrest, induction of tumor cell apoptosis, non-apoptotic cell death, inhibition of tumor angiogenesis, regulation of EMT, and modulation of immune function. This analysis of the multiple synergistic mechanisms underlying Artemisiae Annuae Herba's anti-cancer activity supports its potential as an urgently needed anti-cancer drug and underscores the feasibility of its application in cancer therapies. This potential is further strengthened by early anti-cancer experiments suggesting its wide applicability.

It is important to note that the anti-cancer capabilities of various components of Artemisiae Annuae Herba differ due to their distinct pharmacological activities and mechanisms of action. Sesquiterpenoids, the core components, exert direct anti-cancer effects, demonstrating demonstrable efficacy in inhibiting proliferation and inducing tumor cell death, while coumarins and lignans offer unique advantages in modulating the tumor microenvironment. Furthermore, the auxiliary anti-cancer role of polysaccharide-related components in regulating immune function should not be underestimated.

However, despite the increasing recognition of the anti-cancer effects of Artemisiae Annuae Herba, current research faces several gaps. Existing studies have relied heavily on *in vitro* cell experiments, which, while useful for demonstrating anti-cancer potential in a simplified setting, fail to replicate the complexity of the tumor microenvironment and the host's immune system. This limitation hinders the accurate assessment of the translational potential of Artemisiae Annuae Herba. Furthermore, research has often focused on single-agent artemisinin and its derivatives, neglecting the synergistic regulatory anti-cancer effects of other components, such as polysaccharides and peptides. This isolation of components fragments the complex interactions within Artemisiae Annuae Herba and obscures the potential contributions of regulatory molecules. In addition, many studies do not explicitly state whether the tested component is a pure component or a hybrid product containing small amounts of other regulatory components, leading to inconsistencies in reported anti-cancer efficacy. Finally, the field lacks precision in defining the specific roles of different Artemisiae Annuae Herba components in cancer therapy. Grouping components with diverse mechanisms, such as those primarily targeting tumor cells, inhibiting angiogenesis, or modulating immune function, under the umbrella term “Artemisinin derivatives” impedes clinical translation. Addressing these research gaps requires more comprehensive experimental studies, utilizing animal models, organoid models, and, critically, clinical trials. Increased clinical investigation will elucidate the true efficacy of Artemisiae Annuae Herba in treating cancers, assess the influence of the tumor microenvironment and host immunity on its anti-cancer effects, and identify potential synergistic interactions between its various components through comparative trials. Concurrently, the implementation of additional clinical studies will facilitate the standardized classification and characterization of Artemisiae Annuae Herba components, streamlining research efforts and promoting a more efficient cycle of discovery and clinical translation.

Meanwhile, further development of Artemisiae Annuae Herba in cancer treatment faces several challenges, including guiding ligands related to their desired targets, issues with solubility and drug delivery, potential risks related to drug metabolism, side effects, and safety concerns related to long-term management. Addressing the challenge of ligand targeting through artificially constructed ligands or target precursors could enable Artemisiae Annuae Herba to achieve a breakthrough in precision treatment. This would allow it to specifically identify and target tumor lesions, similar to its anti-malarial activity, potentially facilitating the clinical translation of its cancer therapeutic effects. Overcoming challenges related to drug solubility, delivery, and distribution could also break through the blockade of the strict dosage requirements that currently limit many anti-cancer drugs, enabling standardized regulation of Artemisiae Annuae Herba-based anti-cancer agents. Validating and evaluating sufficient research data in the future could lead to a departure from the current rigid chemotherapy treatment cycles, providing cancer patients with more treatment options and improved time management, ultimately enhancing their quality of life. Furthermore, a deeper understanding of Artemisiae Annuae Herba drug metabolism, coupled with the use of albumin and other related carriers to slow down drug half-life and maintain blood drug concentrations, could provide a solution for long-term and stable anti-cancer needs, positioning it advantageously for clinical translation. Finally, collecting comprehensive experimental data on long-term and high-dose pulse use, and analyzing the corresponding effectiveness, side effects, and potential problems, can effectively prevent subsequent adverse events before it becomes a widely accepted anti-cancer drug.

Artemisiae Annuae Herba has evolved beyond its initial recognition as a specialized anti-malarial drug. Research into its anti-cancer properties is now well established and increasingly recognized. Continued basic research and sustained efforts are actively advancing its practical application in cancer therapies, marking a promising direction for future exploration.

## Figures and Tables

**Table 1:** Anti-cancer components in Artemisiae Annuae Herba and related mechanisms

Component	Molecular structure		Tumor types	Mechanisms	Ref.
Artemisinin	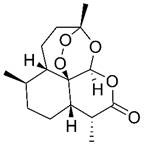		Hematologic	Cell cycle arrest Induced autophagy	336,337
			Esophageal	Cell cycle arrest Regulate EMT Suppress metastasis	338,339
			Gastric	Cell cycle arrest Induce ferroptosis	340,341
			Colorectal	Cell cycle arrest Induce apoptosis	54,205,342
			Lung	Cell cycle arrest Induce apoptosis Inhibit angiogenesis	232,343-345
			Liver	Induce ferroptosis Modulate immune function	346-348
			Pancreas	Induce apoptosis	349
			Breast	Induce apoptosis Induce autophagy Inhibit angiogenesis Modulate immune function	179,192,350-352
			Prostate	Cell cycle arrest Induce apoptosis	127,191,353
				
Dihydroartemisinin	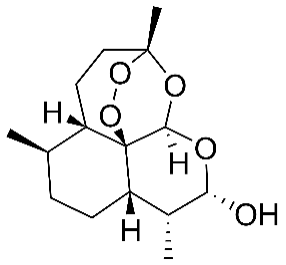		Hematologic	Induce apoptosis Induce ferroptosis	354,355
			Lymphatic	Induce apoptosis Induce autophagy Induce ferroptosis	159,356-358
			Gastric	Cell cycle arrest Induce apoptosis Induce ferroptosis Inhibit angiogenesis Regulate EMT Suppress metastasis	167,359-363
			Colorectal	Induce apoptosis Inhibit angiogenesis Suppress metastasis Modulate immune function	15,364-367
			Lung	Induce apoptosis Induce ferroptosis Inhibit angiogenesis Modulate immune function	245,283,368-370
			Liver	Cell cycle arrest Induce ferroptosis Regulate EMT Modulate immune function	371-374
			Pancreas	Induce apoptosis Induce ferroptosis Modulate immune function	375-381
			Breast	Cell cycle arrest Inhibit angiogenesis Suppress metastasis	243,382,383
			Ovarian	Induce apoptosis Suppress metastasis	384-387
			Prostate	Induce apoptosis	388-390
Artesunate		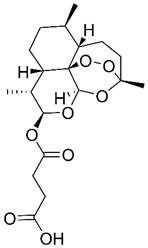	Hematologic	Induce apoptosis Induce autophagy Induce ferroptosis	286,391,392
			Lymphatic	Induce apoptosis Induce autophagy Induce ferroptosis	393-397
			Gastric	Cell cycle arrest Induce apoptosis Inhibit angiogenesis	398-400
			Colorectal	Cell cycle arrest Induce apoptosis Induce ferroptosis Inhibit angiogenesis Suppress metastasis Modulate immune function	165,249,401-403
			Lung	Cell cycle arrest Induce apoptosis Induce ferroptosis Regulate EMT Suppress metastasis	245,318,404-406
			Liver	Induce apoptosis Induce autophagy Induce ferroptosis	16,320,407-409
			Pancreas	Cell cycle arrest Induce ferroptosis	410-413
			Breast	Cell cycle arrest Induce apoptosis Regulate EMT Suppress metastasis	414-418
			Ovarian	Cell cycle arrest Induce apoptosis Induce ferroptosis Inhibit angiogenesis	233,419-423
			Prostate	Cell cycle arrest Induce apoptosis	127,424-426
				
Artemether		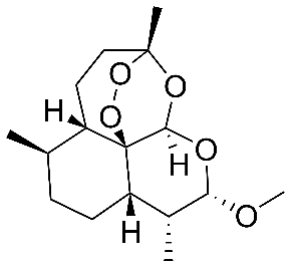	Lymphatic	Cell cycle arrest Induce apoptosis Modulate immune function	427,428
			Lung	Cell cycle arrest Induce apoptosis	429
			Breast	Inhibit angiogenesis	48
				
Artemisitene		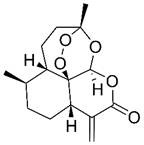	Breast	Induce apoptosis Regulate EMT Suppress metastasis	430,431
Artemisinic acid		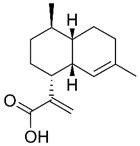	Lung	Induce apoptosis Modulate immune function	72,143
				
Artemisinin B		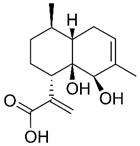	Lung	Induce apoptosis	432
			Liver	Induce apoptosis	143
				
Casticin		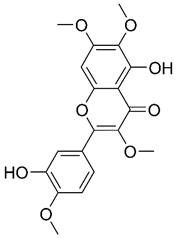	Osteosarcoma	Induce ferroptosis	433
			Melanoma	Suppress metastasis	434
			Glioblastoma	Induce apoptosis Induce autophagy	435,436
				
Chrysosplenol D		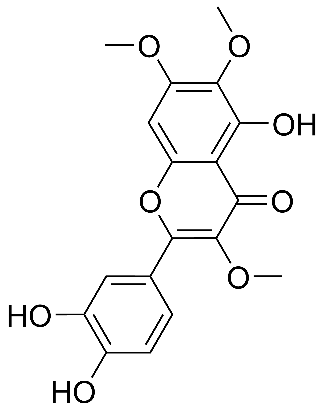	Lung	Induce apoptosis	185
			Breast	Cell cycle arrest Induce apoptosis	57,437
			Prostate	Induce autophagy	438
				
Polysaccharides		/	Liver	Induce apoptosis Suppress metastasis Modulate immune function	171,173
				
Polyphenols		/	Colorectal	Cell cycle arrest Induce apoptosis	282,439,440
			Breast	Regulate EMT Suppress metastasis	186
			Prostate	Cell cycle arrest	440
					

## Abbreviations

3CLpro: 3C-like proteinase
ACTs: artemisinin combination therapies
ADP-ribose: adenosine diphosphate ribose
AFAP1L2: actin filament associated protein 1-like 2
AKT: protein kinase B
ALL: acute lymphoblastic leukemia
ARE: arteether
ARM: artemether
ART: artesunate
BAD: Bcl-2-associated death promoter
BAK: Bcl-2 homologous antagonist killer protein
BAX: Bcl-2-associated X protein
CDKs: cyclin-dependent kinases
DAT: dihydroartemisinin
DHA: dihydroartemisinin
E2F: E2 promoter binding factor
ECM: extracellular matrix
EMT: epithelial-mesenchymal transition
GPX4: glutathione peroxidase 4‌
HIF-1α: hypoxia-inducible factor-1α
HO-1: heme oxygenase-1
HSC70: heat shock protein70
HSP72: human heat shock protein 72
IREs: iron-responsive elements
IRPs: iron regulatory proteins
KDR: kinase insert domain receptor
LC3: microtubule-associated protein 1A/1B-light chain 3
mTOR: mammalian target of rapamycin
MAPK: mitogen-activated protein kinase
MMPs: matrix metalloproteinases
NF-κB: nuclear factor kappa-B‌
NFATc1: nuclear factor of activated T-cells 1
NSCLC: non-small cell lung cancer
OSCC: oral squamous cell carcinoma
pRb: phosphorylated retinoblastoma protein
PI3K: phosphatidylinositol 3-kinase
PKM2: pyruvate kinase muscle isozyme M2
PPARγ: peroxisome proliferator-activated receptor γ
PSA: prostate-specific antigen
PTEN: phosphatase and tensin homolog
RANKL: receptor activator of nuclear factor-κ B ligand‌
ROS: reactive oxygen species
TfR: transferrin receptor
TCM: traditional Chinese medicine
TIMP: tissue inhibitors of metalloproteinase
TLR: toll-like receptors
TSP: thrombospondin
VEGF: vascular endothelial growth factor
